# Aberrant Sensory Gating of the Primary Somatosensory Cortex Contributes to the Motor Circuit Dysfunction in Paroxysmal Kinesigenic Dyskinesia

**DOI:** 10.3389/fneur.2018.00831

**Published:** 2018-10-15

**Authors:** Yo-Tsen Liu, Yi-Chieh Chen, Shang-Yeong Kwan, Chien-Chen Chou, Hsiang-Yu Yu, Der-Jen Yen, Kwong-Kum Liao, Wei-Ta Chen, Yung-Yang Lin, Rou-Shayn Chen, Kang-Yang Jih, Shu-Fen Lu, Yu-Te Wu, Po-Shan Wang, Fu-Jung Hsiao

**Affiliations:** ^1^Department of Neurology, Neurological Institute, Taipei Veterans General Hospital, Taipei, Taiwan; ^2^Faculty of Medicine, National Yang-Ming University, Taipei, Taiwan; ^3^Institute of Brain Science, National Yang-Ming University, Taipei, Taiwan; ^4^Brain Research Center, National Yang-Ming University, Taipei, Taiwan; ^5^Institute of Biophotonics, National Yang-Ming University, Taipei, Taiwan; ^6^Department of Biomedical Imaging and Radiological Sciences, National Yang-Ming University, Taipei, Taiwan; ^7^Department of Neurology, Chang Gung Memorial Hospital, Chang Gung University, Taoyuan, Taiwan; ^8^College of Medicine, Chang Gung University, Taoyuan, Taiwan; ^9^Department of Neurology, Taipei Municipal Gan-Dau Hospital, Taipei, Taiwan; ^10^Department of Critical Care Medicine, Taipei Veterans General Hospital, Taipei, Taiwan

**Keywords:** paroxysmal kinesigenic dyskinesia, magnetoencephalagraphy, gamma oscillation, primary somatosensory cortex, *PRRT2*, sensory gating

## Abstract

Paroxysmal kinesigenic dyskinesia (PKD) is conventionally regarded as a movement disorder (MD) and characterized by episodic hyperkinesia by sudden movements. However, patients of PKD often have sensory aura and respond excellently to antiepileptic agents. *PRRT2* mutations, the most common genetic etiology of PKD, could cause epilepsy syndromes as well. Standing in the twilight zone between MDs and epilepsy, the pathogenesis of PKD is unclear. Gamma oscillations arise from the inhibitory interneurons which are crucial in the thalamocortical circuits. The role of synchronized gamma oscillations in sensory gating is an important mechanism of automatic cortical inhibition. The patterns of gamma oscillations have been used to characterize neurophysiological features of many neurological diseases, including epilepsy and MDs. This study was aimed to investigate the features of gamma synchronizations in PKD. In the paired-pulse electrical-stimulation task, we recorded the magnetoencephalographic data with distributed source modeling and time-frequency analysis in 19 patients of newly-diagnosed PKD without receiving pharmacotherapy and 18 healthy controls. In combination with the magnetic resonance imaging, the source of gamma oscillations was localized in the primary somatosensory cortex. Somatosensory evoked fields of PKD patients had a reduced peak frequency (*p* < 0.001 for the first and the second response) and a prolonged peak latency (the first response *p* = 0.02, the second response *p* = 0.002), indicating the synchronization of gamma oscillation is significantly attenuated. The power ratio between two responses was much higher in the PKD group (*p* = 0.013), indicating the incompetence of activity suppression. Aberrant gamma synchronizations revealed the defective sensory gating of the somatosensory area contributes the pathogenesis of PKD. Our findings documented disinhibited cortical function is a pathomechanism common to PKD and epilepsy, thus rationalized the clinical overlaps of these two diseases and the therapeutic effect of antiepileptic agents for PKD. There is a greater reduction of the peak gamma frequency in *PRRT2*-related PKD than the non-*PRRT* PKD group (*p* = 0.028 for the first response, *p* = 0.004 for the second response). Loss-of-function *PRRT2* mutations could lead to synaptic dysfunction. The disinhibiton change on neurophysiology reflected the impacts of *PRRT2* mutations on human neurophysiology.

## Introduction

Magnetoencephalography (MEG) is a powerful tool because it is not only competent in localizing and measuring the cortical neuronal activities, but also able to show the temporal dynamic changes and the reaction synchronicity between different functional areas (1). Brain oscillations at different frequencies detected via MEG represent the neural networking of various brain functions. Oscillations in the gamma frequency band (30 to 100 Hz) are driven by “bottom-up” brain processing which involves the activation of lower brain centers by sensory events such that the information moves to higher centers to promote perception (2, 3). The role of synchronized gamma oscillations in sensory gating, binding of information from various regions into a united whole, has been recognized as an important mechanism of automatic cortical inhibition (4, 5). The patterns of gamma oscillations, representing cortical synchronization, have been used to characterize the electrophysiologic features of various neurological diseases. For instance, gamma oscillatory epileptic activity in (between 60 and 100 Hz) in human EEG have been known for long to spatially coincide with the location of epileptic seizures and therefore called high-frequency epileptiform oscillations (6, 7). In movement disorders (MDs), the broadband gamma (50–200 Hz) power in the primary motor cortex is greater in both isolated dystonia and Parkinson disease (PD), suggesting a physiologic overlap with respect to the reduction of motor cortical synchronization (8). On the other hand, dystonic patients had longer somatosensory temporal discrimination thresholds, reduced suppression of cortical and subcortical paired-pulse somatosensory evoked potentials, less spatial inhibition of simultaneous somatosensory evoked potentials (9).

Paroxysmal kinesigenic dyskinesia (PKD) is a group of diseases characterized by episodic involuntary movements triggered by sudden movements, like initiation of standing, walking, or running. Attacks may consist of dystonic, choreatic, and mixed symptoms. PKD is conventionally regarded as a movement disorder involving substantia nigra and basal ganglia (10, 11). However, PKD also shared clinical presentations with epilepsy. Patients quite often have sensory aura like numbness or tingling sensation in the limbs and have excellent response to antiepileptic agents such as carbamazepine and phenytoin (12–17). Recent advancement of molecular genetics identified *PRRT2* mutations are the most common genetic etiology of PKD, which could cause epilepsy syndromes as well (17–23). Standing in the twilight zone between movement disorders and epilepsy, the pathogenesis of PKD is still unclear. Our previous MEG studies have identified cortical involvement in PKD, including the motor (24, 25) and somatosensory cortex (26), which pointed to the deficits of cortical inhibition may be the underlying neurophysiological mechanism of this disease. Recently, we further reported that the inhibitory function of stimulus-evoked somatosensory gating activities was significantly correlated with movement-related sensorimotor oscillatory responses (27). This logically brought the idea that the primary somatosensory cortex is a critical region for PKD. There are strong connections between basal ganglia, motor areas and the primary somatosensory areas (28). To clarify the relationship between abnormality of motor response and deficiency of sensory processing in PKD, it is mandatory to further characterize the pattern of gamma synchronization in the somatosensory area.

This study was aimed to explore the features of gamma synchronizations in PKD with the attempt to address the alternations of brain sensory and motor networks of the disease. We totally recruited 19 patients of newly-diagnosed PKD, 5 with a pathogenic *PRRT2* mutation, and 18 healthy controls (HC). Distinguished from previous MEG studies for PKD, all our patients were evaluated before receiving any pharmacotherapy so that the medication effects could be avoided. In addition, this study was based on a relative homogenous phenotype as all of the recruited patients presented by paroxysmal dystonia but no chorea. This is also the first study to explore the impacts of *PRRT2* mutations on human neurophysiology.

## Materials and methods

### Ethics approval

This work was carried out with informed consents obtained from the participants and the ethics approval from the institutional review board of Taipei Veterans General Hospital (IRB 2015-08-001B).

### Patients

This study recruited nineteen patients newly diagnosed as PKD according to the criteria proposed by Bruno et al. (13). All patients were evaluated before receiving any pharmacotherapy for PKD. Eighteen age- and gender-matched healthy controls (HC) were also recruited. All participants were right-handed and presented by normal physical and neurologic examinations.

### Genetic analysis of *PRRT2* mutations

Genomic DNA was extracted from the white blood cells in the peripheral blood with standard protocols. All 19 PKD patients received mutation analysis of the coding exons and flanking introns of the *PRRT2* gene (NM_145239.2) by Sanger sequencing. The detected mutations were validated via repeated sequencing of sense and antisense strands of the amplicons. The pathogenicity determination took references of the following public genome dababase: the Exome Aggregation Consortium Sequencing Project genome database (ExAC; http://exac.broadinstitute.org), dbSNP (https://www.ncbi.nlm.nih.gov/snp/), 1000 Genomes Project (www.1000genomes.org), the National Heart, Lung, and Blood Institute Exome Sequencing Variant database (http://evs.gs.washington.edu/EVS/) and Taiwan Biobank (https://taiwanview.twbiobank.org.tw).

### MEG recording

Median nerve stimulation was eperformed to provoke somatosensory evoked potentials (SEPs) and fields, which is a standard investigating paradigm using multichannel MEG simultaneous recordings (29, 30).

Each subject received median-nerve electrical stimulation at the right wrist by an electrical stimulator (Konstant-Strom Stimulator) using the paired-pulse paradigm, which means two identical electrical pulses were delivered with short time interval between them. Each pulse of the paired stimulations was constant-current square-wave with the width of 0.2 ms, inter-stimulus interval (ISI) of 500 ms and inter-pair interval of 8 s, as reported in our previous studies (26, 31). The stimulus intensity was individually set at 20% above the motor threshold for eliciting a visible twitch of the abductor pollicis brevis muscle. There was no significant difference of the stimulus intensity between the two groups (HC: 4.16 ± 0.11 milliamperes (mA); PKD: 4.31 ± 0.15 mA, *p* > 0.05).

Somatosensory evoked fields (SEFs) for paired electrical stimulation were recorded by using a whole-scalp 306-channel MEG (VectorviewTM, Elekta Neuromag, Helsinki, Finland), which is composed of 102 identical triple sensor elements. Each sensor element consisted of two orthogonal planar gradiometers and one magnetometer. In the following analysis, the neuromagnetic activities from magnetometers were discarded for low signal-to-noise ratio (1). To precisely localize the cortical activities, four coils stand for the head position were placed on the subject's scalp, and their positions in the head coordinate frame specified by the nasion and two pre-auricular points were measured with a 3-dimension digitizer using Cartesian coordinates. There were 50 additional scalp points digitized, and these landmarks and points of the head position allowed for further registration of the MEG and MRI coordinate systems. Two electrodes attached above and below one eye were used to simultaneously detect the electrooculography activities. During the recordings, the subjects sat comfortably with the head supported against the helmet of the neuromagnetometer.

The sampling rate was 600 Hz. The epoch was collected with a 1400 ms time window including a 500 ms baseline before the first stimulus onset, of which the epoch ranged from −500 to 900 ms and the first and second electrical stimulations were at 0 and 500 ms, respectively. Epochs contaminated by prominent electro-oculogram signals [> 300 microvolts (μV)] or MEG artifacts (> 3000 fT/cm) were automatically excluded from further analysis. At least one hundred artifact-free data epochs, in which included the first and second SEF responses to the paired stimulations, were recorded for further analyses.

### MEG data analysis

Somatosensory gamma oscillations were evaluated using the SEF responses and transformed into time-frequency domain using Morlet wavelet analysis. A set of wavelets was used with frequencies ranging from 1 to 100 Hz in 1 Hz steps. The default wavelet was set at 1 Hz and 3 seconds for central frequency and time resolution, respectively. Time-frequency spectral power of the SEF responses was averaged across all epochs. At each time-frequency bin was normalized as the percentage change of spectral power relative to the mean power in a reference period between −500 and −100 ms. In the present study, we extracted the prominent channel with the largest SEF responses and calculated the peak power, frequency and latency of gamma oscillation following the first and second electric stimulation at the frequency range over 30 ~ 100 Hz.

To localize the gamma oscillation, the SEF responses were analyzed with a distributed source modeling using weighted minimum norm estimates analysis. The forward model was calculated from the MRI-derived surface model of each participant's brain to describe the signal pattern generated by a unit dipole at each allowed location on the surface. Brain MRIs were acquired by using a 3T MR system (Siemens Magnetom Tim Trio). The surface model was reconstructed from the T1-weighted structural volumetric images (BrainVISA 4.0.2, http://brainvisa.info). The inverse operator of minimum norm estimates analysis was calculated with noise covariance derived from baseline period (−500 ~ −100 ms) and regularization parameter (λ^2^ = 0.33). Finally, the cortical source activities were obtained and were further analyzed to map the oscillatory activities onto the cortical surface and MRI using the time-frequency power analysis at specific frequency range as mentioned above. The MEG data analysis was performed with Brainstorm (32), which is a documented program freely available online under the GNU general public license (http://neuroimage.usc.edu/brainstorm).

### Statistical analysis

The peak power, frequency and latency of gamma oscillation for the first and second responses were compared between the HC group and the PKD group by nonparametric Mann-Whitney U test, as well as between the PKD patients with and without a *PRRT2* mutation. Group difference of the gamma power ratio of the second response to the first response was also tested. Moreover, the chi-square and logistic regression with bootstrap technique were performed to relate the genetic factors to the gamma oscillations. All hypotheses were constructed as two-tailed. The *p*-value < 0.05 was considered statistically significant.

## Results

### Clinical features and genetic diagnosis of the patients

The demographic and clinical features were summarized in Table 1. The average age of examination (AOE) was 24.4 ± 6.0 years for PKD patients (male: female = 15: 4) and 28.4 ± 3.6 year for the HC (male: female = 16: 2), without significant difference between two groups. All patients presented by paroxysmal dystonia, but no chorea or ballism. They were examined before receiving any medication for PKD and the attack frequencies and durations were referred to the conditions of the drug naïve status. There are three pathogenic *PRRT2* mutations identified in five unrelated PKD patients: c.649_650insC:p.Arg217ProfsX, c.649_650delC:R217EfsX and c.971delG:p.Gly324GlufsX.

**Table 1:** Clinical features and genetic diagnosis of the patients of PKD.

**Patient No**.	***PRRT2* mutation**	**Gender**	**AOE**	**AON**	**DD**	**Frequency/month**	**Duration (second)**	**Family history**
1	R217EfsX	M	38	9	29	10–20	10	N
2	R217PfsX	M	28	11	17	20–30	10–15	N
3	R217PfsX	F	24	16	8	20–30	10–15	Y
4	R217PfsX	M	19	12	7	20–30	5–10	Y
5	G324EfsX	M	26	12	14	20–30	5–10	Y
6	N	M	18	13	5	5–10	< 5	N
7	N	M	25	10	15	20–30	5–10	N
8	N	M	19	16	3	20–30	< 5	N
9	N	M	18	13	5	20–30	5–10	Y
10	N	M	23	14	9	20–30	5–10	N
11	N	F	17	12	5	< 5	< 5	Y
12	N	M	27	14	13	20–30	20–30	N
13	N	M	23	10	13	20–30	5–10	Y
14	N	F	17	11	6	20–30	5–10	Y
15	N	M	26	10	16	10–20	5–10	N
16	N	F	38	20	18	10–20	5–10	N
17	N	M	27	10	17	5–10	5–10	N
18	N	M	25	13	12	20–30	20–30	N
19	N	M	25	17	8	< 5	20–30	N
*PRRT2*-related PKD			27.0 ± 7.0	12.0 ± 7.9	15.0 ± 8.9	/	/	60%
Non-*PRRT2* PKD			23.4 ± 5.6	13.1 ± 3.0	10.4 ± 5.1	/	/	28.6%

*AOE, Age of examination (year); AON, Age of onset (year); DD, disease duration (AOE-AON, year); Duration: duration of each attack; N, no; Y, yes*.

### Aberrant gamma synchronizations of the primary somatosensory cortex in PKD

**Figure 1:**
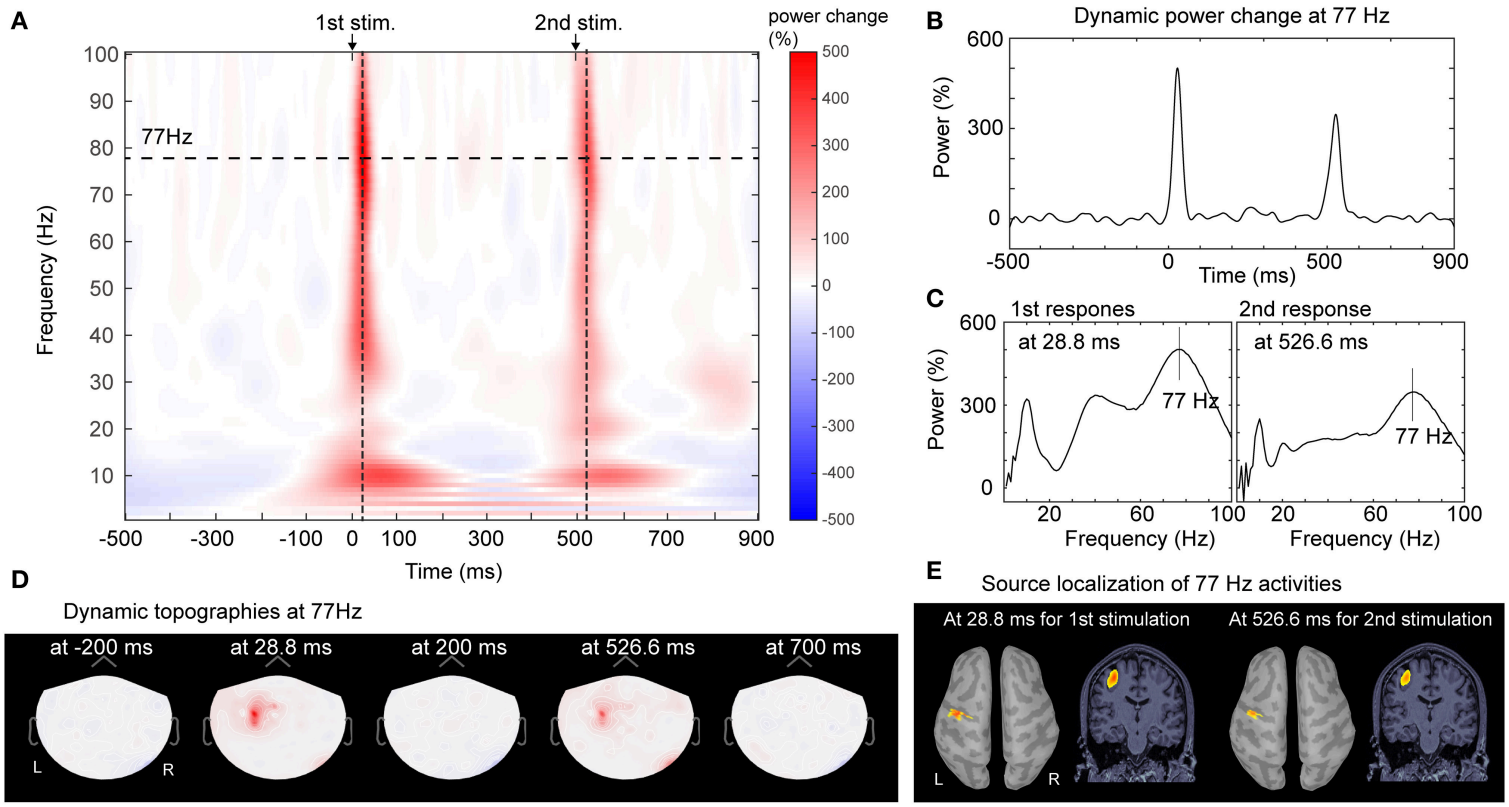
Analysis of gamma oscillations of SEF responses. The time-frequency representation of cortical activities from one channel in response of paired-pulse electrical stimulation from Control No. 1 was shown as the example. **(A)** Prominent power increase was observed after the first and second electric stimulations, especially at the frequency bands of 5~20 Hz and 30~100 Hz. In the frequency range of gamma oscillation, the peak frequency was 77 Hz for both responses. **(B)** The time-varying dynamic power change of the peak gamma activities (at 77 Hz) in the time window of−500~900 ms (long-dashed line in 1A) exhibited the peak latencies at 28.8 and 526.6 ms for first and second response respectively. **(C)** The power spectrums of the maximal power increase located at 77 Hz for both the first and second stimulation. **(D)** The topographic distributions of peak gamma oscillation showed the temporal-dynamic and spatial-specific cortical activation. Synchronized gamma increase was elicited contralaterally and locally. **(E)** Localization of gamma oscillation at peak frequencies and latencies for the first and second stimulations were mapped onto the primary somatosensory area on the reconstructed cortical surface of brain. MRI. L, left; R, right; 1st, the first; 2nd, the second.

**Figure 2:**
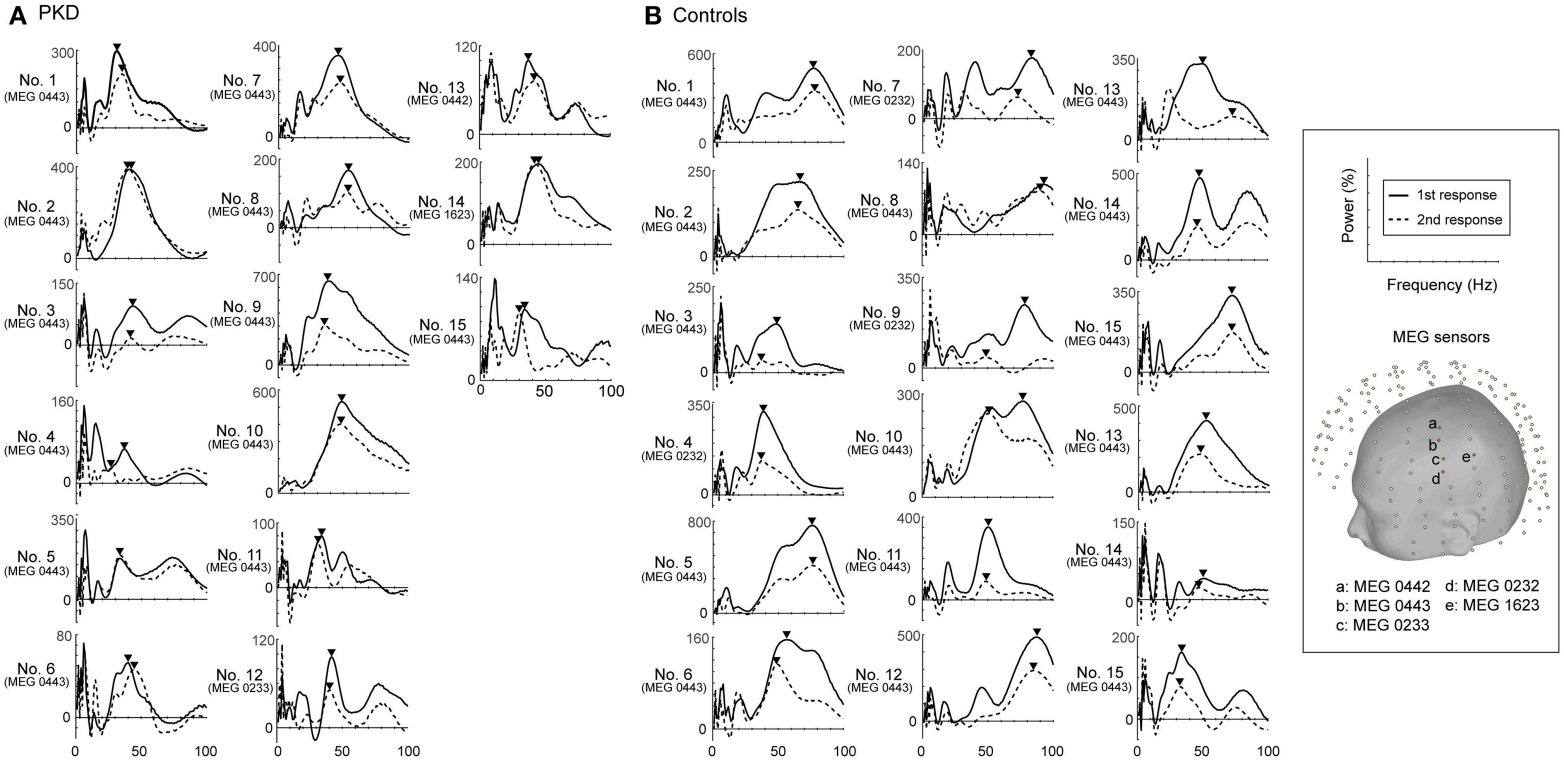
Power spectrums of gamma synchronization. The power spectrum (0-100 Hz) at the peak latencies of first and second responses for each of the 19 PKD patients and 18 controls were listed. The peak frequencies in the gamma frequency range (30~100 Hz) were obtained and indicated with inverted-triangle symbol. **(A)** In all PKD patients, the peak frequency ranged between 30~50 Hz. **(B)** In the majority of the control subjects (16/18, except No. 4 and No.18), the peak gamma frequency was at 45~90 Hz.

**Figure 3:**
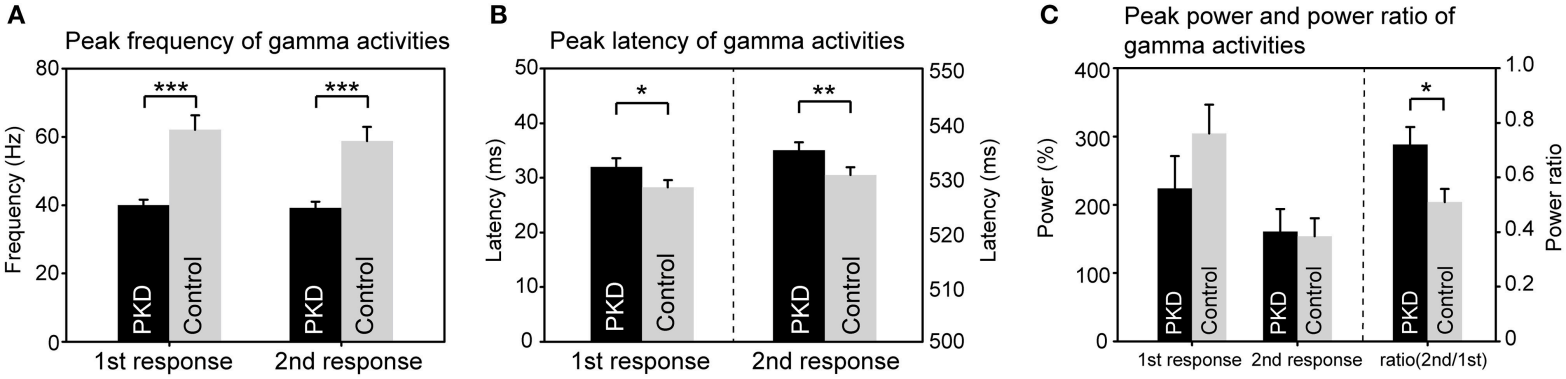
Group difference of gamma synchronization between PKD and the healthy controls (HC). **(A)** The peak frequency of gamma oscillation in the PKD group was much smaller than that in the HCl group (for the first response, PKD: HC = 40.1 ± 1.5 Hz: 62.1 ± 4.2 Hz, *p* < 0.001; for the second response, PKD: HC = 39.2 ± 1.8 Hz: 58.8 ± 4.1 Hz, *p* < 0.001). **(B)** The peak latency of gamma oscillation was significantly delayed in PKD as compared with the HC (for the first response, PKD: HC = 31.99 ± 1.59 ms: 28.26 ± 1.33 ms, *p* = 0.02; for the second response, PKD: HC = 535.04 ± 1.46 ms: 530.52 ± 1.43 ms, *p* = 0.002). **(C)** There was no group effect for the power of gamma. The power ratio of the second response to the first response was higher in PKD than in HC (PKD: HC = 0.72 ± 0.064: 0.51 ± 0.048, *p* = 0.013). ***p < 0.001, **p < 0.01, *p < 0.05.

The record from HC No.1 was shown as an example (Figure 1). Gamma oscillations of SEF exhibit a temporal-dynamic change with a suppressive effect on external stimulation (Figure 1A). Normally the second response is smaller than the first response, representing an essential sensory gating of the brain (Figure 1B). Using MEG and MRI together, gamma oscillations were proved to be spatially specific to the somatosensory cortex (Figure 1E). The power spectrum of somatosensory gamma oscillations of all patients and HC participants were shown in Figure 2. The range of peak frequency was smaller in patients with PKD (30~50 Hz, Figure 2A) than in the HC (45~90 Hz, Figure 2B). In comparison with HC, the reduction of gamma peak frequency in PKD was quite substantial (*p* < 0.001 for both the first and the second response, Figure 3A). The above finding indicated there was aberrant neuronal synchronization in the primary somatosensory cortex in patients with PKD. The patients also had significantly delayed peak latencies of gamma oscillations (*p* = 0.02 for the first response and *p* = 0.002 for the second response, Figure 3B). Gamma latency is correlated with SEF N20 and P35 latency response. The analysis of our results showed a prolonged peak latency in average in PKD patients (higher than that in HC). This means that the affected individuals with dyskinesia have slower gamma oscillation responses, and again, reflected the attenuated synchronization of gamma oscillations. We also measured the power ratio to observe the inhibitory function of gamma oscillations. The power ratio is defined as the proportion of the gamma power for second stimulation to that for first one, similar to measurements in primary somatosensory cortex using gating ratio (2nd SEF amplitude/1st SEF amplitude). Therefore, the significantly higher power ratio in the PKD group (*p* = 0.013, Figure 3C) means that the ability to inhibit the response for the second stimulation was lost, indicating the incompetence of activity suppression but not the capability of spent energy.

### A greater reduction of the peak gamma frequency related to *PRRT2* mutations

**Figure 4:**
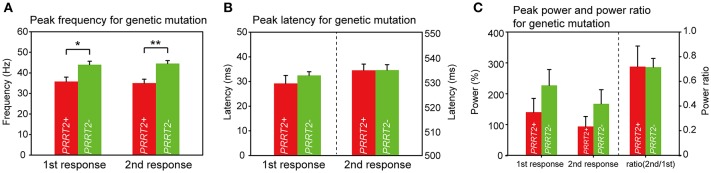
Group difference of gamma synchronizations between *PRRT2*-related PKD and non-*PRRT2* PKD. **(A)** The PKD patients with an identified *PRRT2* pathological mutation (*n* = 5) had lower evoked peak gamma frequencies than those patients without a *PRRT2* mutation (*n* = 9) (for the first response, *PRRT2*+ PKD: *PRRT2*- PKD = 35.8 ± 2.1 Hz: 44 ± 1.7 Hz, *p* = 0.028; for the second response, *PRRT2*+ PKD: *PRRT2*- PKD = 35 ± 1.9 Hz: 44.5 ± 1.4 Hz, *p* = 0.004). **(B,C)** There was no difference of the peak latency, peak power and power ratio of gamma activities between *PRRT2*+ PKD and *PRRT2*- PKD. *PRRT2*+ PKD = *PRRT2*-related PKD, *PRRT2*- PKD = non-*PRRT2* related PKD, **p < 0.01, *p < 0.05.

We then looked for the group difference of gamma synchronizations between *PRRT2*-related PKD and non-*PRRT2* PKD. As listed in Table 1, except for a higher proportion of the positive family history in *PRRT2*-related PKD (*PRRT2*-related PKD: 60%, non-*PRRT2* PKD: 28.6%), the mean AOE, age of onset (AON) and disease duration (DD) had no significant difference in patients with or without a pathogenic *PRRT2* mutation. Although the peak gamma frequencies in both *PRRT2*-related PKD and non-PRRT2 PKD were significantly lower than HC, it was noticeable that the peak gamma frequency had a greater reduction in *PRRT2*-related PKD as compared with non-*PRRT2* PKD (*p* = 0.028 for the first response and *p* = 0.004 for the second response, Figure 4A). As for chi-square test, a significantly lower peak gamma frequency was associated with a *PRRT2* mutation in PKD (*p* = 0.021 for the second response). Moreover, using logistic regression analysis, significant relationships were observed between a *PRRT2* mutation and peak gamma frequency (odds ratio: 1.5 for first response and 2.1 for second one; all p < 0.01 with bootstrap technique). The differences of the peak latency (Figure 4B), peak power and power ratio (Figure 4C) of gamma activities between the two groups did not reach a significant level.

## Discussion

This study identified that patients of PKD have aberrant synchronization of gamma oscillations in the primary somatosensory cortex. Compared with HC, the PKD group had a substantial reduced peak frequency, showing a reduced synchronization of neuron oscillations. The prolonged peak latency in PKD (higher than that in HC) means that the affected individuals with dyskinesia have slower gamma oscillation responses, and again, reflected the aberrant synchronization of gamma oscillations. The higher power ratio in the PKD group further indicates the incompetence of activity suppression. All together, these findings indicate that somatosensory sensory gating was defective in PKD.

The involvement of primary somatosensory area in current study is in line with several functional MRI studies for PKD (33–35). As the primary somatic sensory cortex is responsible for processing tactile information and receives the bulk of the thalamocortical projections from the sensory input fields (36), abnormal somatosensory sensory gaiting explains clinically why changing body postures can trigger the episodic symptoms in PKD. The sensory aura in PKD patients may be another semiology in associated with the dysfunction of sensory procession.

Our group has previously identified decreased postmovement inhibition of the motor cortex and altered theta synchronization in the somatosensory areas between both hemispheres in patient with PKD (24, 26, 37). Added by attenuated somatosensory sensory gating identified in current study, these neurophysiological changes together addressed the nature of PKD as a network disorder which involves not only motor functions but also the sensory processing pathways.

The disinhibitory deviation of neuronal networks in PKD could be supported by evidence at the molecular level. Gamma oscillations represent a specific frequency code of GABA (gamma-aminobutyric acid)-ergic inhibitory interneurons which are crucial in the sensorimotor integration in the corticocortical and thalamocortical circuits (38, 39). Gamma frequency has been correlated with GABA concentration or acceptor density in the magnetic resonance spectroscopy (MRS) or Flumazenil-PET studies (40–42). Through the link of GABAergic neurotransmission and gamma oscillations, it is reasonable to postulate that aberrant somatosensory gamma frequency in PKD is a consequence of the imbalance between inhibitory and excitatory neuronal transmission. This is also the core pathomechanism of epileptogenesis. Aberrant gamma oscillations point to a pathophysiological change underlying both epilepsy and PKD and thus help explain the overlaps of clinical manifestations between these two diseases. GABAergic disinhibition is also an important target for pharmacotherapy. Antiepileptic drugs, like carbamazepine and phenytoin, have shown interactions with the GABA receptors through various mechanisms (16, 26, 43). The modeling effect on GABAergic gamma oscillations provides the rationale of the excellent treatment response of PKD to these antiepileptic drugs.

Noticeably, our patients who carried a pathogenic *PRRT2* mutation had a much more decreased peak gamma frequency. PRRT2 is a component of the AMPAR (α-amino-3-hydroxy-5-methyl-4-isoxazolepropionic acid receptor) complex and believed to be involved in synaptic transmission of diverse signaling molecules, including glutamate (44–48). We have previously documented that these loss-of-function *PRRT2* mutations carried by our patients could lead to synaptic dysfunction (49). As the peak value of gamma frequency is closely related to the GABA concentration in corresponding brain areas (50), our finding suggested that *PRRT2* mutations would lead to synaptic dysfunction and then indirectly interrupts GABAergic neurotransmission.

Abnormalities in synchronized gamma oscillatory neuronal activity in the network comprising the basal ganglia, thalamus, and motor cortices are also closely related to the onset of MDs. For example, the broadband gamma power is increased in PD patients with peak-dose dyskinesia (8, 51, 52). Patients with isolated dystonia had enhanced somatosensory temporal discrimination threshold and reduced suppression of paired-pulse SEPs (8, 9), which were also observed in our patients who all presenting by paroxysmal dystonia. In the hamster model with the phenotype of dystonia, maturation of striatal GABAergic interneuron is delayed (53). Reduced functional connectivity of somatosensory network is involved in the pathomechanism of writer's cramp as well (54). Therefore, somatosensory gamma oscillations may be a featuring marker of dystonia and, furthermore, a potential modulation target for therapy.

All of our patients were evaluated before receiving any medications. Unlike previous MEG studies in PKD, this work took advantage on getting rid of the medication effects and is able to reveal the nature of the disease itself. The SEF responses were elicited from the stimulation of the dominant hand (right side) in all participants concerning the hemispheric asymmetry in human somatosensory cortical activation (55). Therefore, four patients received the electrical stimulation in the unaffected side. This may not be a critical issue as our in-house data showed no significant difference in all measurements of gamma synchronization between the responses elicited from the affected and unaffected side in these patients. This study did not reveal any obvious influence of the gender on the gamma oscillations as there was no significant difference in all measurements between male and female patients. Further studies recruiting more female participants may be helpful in observing the gender effect.

## Conclusion

This study identified that aberrant gamma synchronization, representing the attenuation of automatic cortical inhibition on sensory gating, is a neurophysiological feature of PKD. Interrupted sensory gaiting, together with reduced postmovement inhibition identified by our previous works, documents the nature of PKD a network disorder which involves not only motor functions but also the sensory processing pathways. The link of gamma oscillations with GABAergic inhibitory neurotransmission discloses a common pathogenesis underlying PKD and epilepsy and makes the rationale of pharmacotherapy by antiepileptic drugs. Our results also stress the importance to identify the PKD patients with a *PRRT2* mutation because they can beneficial substantially by treatment targeting to GABA deregulation and synaptic dysfunction. Our study highlighted a key role of the primary somatosensory cortex in the pathogenesis of PKD. The concept may apply to dystonia and other MDs and provide a potential targets for modulation in the future.

## Author contributions

Y-TL contributed to the conception and design of the study, acquisition of data, analysis and interpretation of data, drafting of the article and revision of its content. Y-CC, S-YK, C-CC, H-YY, D-JY, K-KL, W-TC, Y-YL, R-SC, K-YJ, S-FL, Y-TW, and P-SW contributed to acquisition of data, drafting of the article, and revision its content. F-JH contributed to the conception and design of the study, acquisition of data, analysis and interpretation of data, drafting of the article and revision its content, and final approval of the version to be submitted.

### Conflict of interest statement

The authors declare that the research was conducted in the absence of any commercial or financial relationships that could be construed as a potential conflict of interest.

## Abbreviations

GABA: gamma-aminobutyric acid
HC: healthy control
MEG: magnetoencephalography
MRI: magnetic resonance imaging
MD: movement disorder
PD: Parkinson disease
PKD: paroxysmal kinesigenic dyskinesia
*PRRT2*: the proline rich transmembrane protein 2 gene
SEF: somatosensory evoked field.
